# Human and ecological determinants of the spatial structure of local breed diversity

**DOI:** 10.1038/s41598-018-24641-3

**Published:** 2018-04-24

**Authors:** Victor J. Colino-Rabanal, Roberto Rodríguez-Díaz, María José Blanco-Villegas, Salvador J. Peris, Miguel Lizana

**Affiliations:** 1 Area of Zoology, Department of Animal Biology, Parasitology, Ecology, Edaphology and Agronomic Chemistry, University of Salamanca, Campus Miguel de Unamuno, 37071 Salamanca, Spain; 2 Area of Physical Anthropology, Department of Animal Biology, Parasitology, Ecology, Edaphology and Agronomic Chemistry, University of Salamanca, Campus Miguel de Unamuno, 37071 Salamanca, Spain

## Abstract

Since domestication, a large number of livestock breeds adapted to local conditions have been created by natural and artificial selection, representing one of the most powerful ways in which human groups have constructed niches to meet their need. Although many authors have described local breeds as the result of culturally and environmentally mediated processes, this study, located in mainland Spain, is the first aimed at identifying and quantifying the environmental and human contributions to the spatial structure of local breed diversity, which we refer to as livestock niche. We found that the more similar two provinces were in terms of human population, ecological characteristics, historical ties, and geographic distance, the more similar the composition of local breeds in their territories. Isolation by human population distance showed the strongest effect, followed by isolation by the environment, thus supporting the view of livestock niche as a socio-cultural product adapted to the local environment, in whose construction humans make good use of their ecological and cultural inheritances. These findings provide a useful framework to understand and to envisage the effects of climate change and globalization on local breeds and their livestock niches.

## Introduction

Livestock diversity is the result of a two-stage process: first the domestication, then the breed differentiation. Both stages are evolutionary and cultural processes that involve genetic changes^1^. Domestication can be regarded as a co-evolving mutualism between humans and animals^2,3^ that evolve in the context of dynamic niche construction^4^. Humans started to domesticate animals at various separate world areas independently more than 10,000 years ago^5^, which transformed the socio-economic situation of most populations^6^. Domestication is followed by differentiation. Breed differentiation is a dynamic process in which man selects a group of animals on the basis of some definable and identifiable external characteristics, which are inheritable and distinguish by a visual appraisal from other groups of animals within the same species^7,8^. The maintenance of a breed over time is achieved through reproductive isolation, that is, mating occurs within the groups but not usually between them^9,10^. Factors such as geographic isolation, ecological characteristics, historical processes, and human geography play a role in explaining this genetic isolation^11,12^. Thus, both natural and artificial selection are involved in breed diversification and give rise to the livestock’s genetic diversity. Each breed has been adapting for many years to the specific environmental conditions of the geographical region in which it is present^13,14^. At the same time, breed differentiation can be considered as a cultural process^1,15^ in which the artificial choice of breed characters is dictated by economic, cultural, aesthetic, or ritual reasons^7^. Modern genetic analysis contributes to the unraveling of the adaptive events entailed in the formation and expansion of the different breeds and the relationships among them^16–18^. Breed genomes show the footprints of long and complex processes, including migration, expansion, admixture, or challenges related to disease and climate change^19,20^. It is possible to detect genetic signatures of the natural and human driven-selection^21^ in cattle^22^, goats, and sheep^23–25^, and pigs^26^.

The result of culturally- and environmentally-mediated breed differentiation processes is a wide range of breeds throughout the world. The estimated number of breeds of livestock worldwide is 8,774^27^. Out of this, 7,718 are local breeds, those which *“have largely developed through adaptation to the natural environment and traditional production system in which it has been raised”*^28^. The ratio of these local breeds has gradually decreased due to the introduction and expansion of commercial breeds^29,30^. Thus, 7.4% (647 breeds) are classified as extinct and another 9.2% (811) as at risk^27^.

Since local breeds can be described as a cultural expression of adaptation to local environmental conditions, they are a relevant form of cultural niche construction. Indeed, together with traditional crops, local breeds are the most powerful way in which each human group has constructed its specific niche, a mixture of cultural and environmental factors. According to the niche-construction theory^31,32^, humans engineer environments to ensure their livelihood. With this aim, those traits that increase the productivity and foreseeability of domesticated species have been selected through generations. The resulting locally-adapted breeds, in turn, also play an active role in ecosystem engineering and niche alteration. Both humans and domesticates benefit from this relationship^4,33–38^. This intergenerational mutualism persists and evolves over time by the generational transmission of the traditional ecological knowledge, progressively accumulating an important ecological and cultural inheritance that conducts and feedbacks the relationship^38,39^. Following the use of the term “niche” associated with niche construction theory^34^, in this study the term “livestock niche” has been coined to refer to that socio-cultural niche constructed by the relationship between humans and domesticated species mediated by cultural and ecological inheritance. The composition of local breeds constitutes the main expression of each of these livestock niches.

In order to increase our understanding of the processes of livestock niche construction and diversification, it is necessary to examine and quantify, in detail, the factors involved, with particular regard to the human and environmental variables. In this respect, studies are scarce or inexistent, and there is still much potential for further research. The comparison of the spatial structuring of explaining factors and the composition of local breeds would provide understanding about the interrelations between them and their relative importance in livestock niche characterization. Since local breeds can be considered as human artifacts and respond to cultural processes, any kind of subdivision in human groups as ethnicity, language, administrative borders, etc. might lead to changes in the livestock niche, for example by imposing mating constraints^1,40,41^. The obvious test is whether there is any parallel between the distances between human populations and between their livestock breeds. It would be expected that the spatial structure of local breed diversity would be connected with human migration patterns and interchanges among neighboring human populations. In addition, apart from limitations to human population flows, political borders may also impose a number of restrictions to livestock fluxes between territories. Comparative linguistics is also expected to contribute to our understanding of the variations in livestock niches^1^. Naturally, together with the human background, the environmental conditions must be taken into account to understand spatial changes in livestock niches and local breed distributions.

This study is the first aimed at identifying and quantifying the environmental and human contributions to livestock niche formation. It is hypothesized that the more similar two zones are in relation to the environment, human population, and history, the more similar their local breeds will be. To test this hypothesis, we quantified the spatial patterns of the human population, the ecological characteristics, and the historical ties of the 47 provinces of mainland Spain and compared them with the spatial structure of the local breed diversity. Euclidean geographical distances were also incorporated into the analyses to rule out the possibility that any correspondence was explained exclusively due to a model of isolation by distance.

## Results

According to the Mantel tests (Table 1), the correlation between the distance matrix of local breeds and each of the other distance matrices is significant in all cases (p < 0.001). When the geographical distance was controlled, the partial Mantel tests also revealed a correlation between local breeds and human population distances, breeds and ecological distances, and also between breeds and historical distances (Table 1).

**Table 1:** (a) Results of the Mantel test (r_M_ and p-values) of the distance matrix of local breeds and the human population, ecological distances, historical and geographical distances.

**a**.	Distance matrix of local breeds	
Mantel test	r_M_	p-value
*Human population distance*	0.499	<0.001
*Ecological distance*	0.462	<0.001
*Historical distance*	0.392	<0.001
*Geographic distance*	0.501	<0.001
**b**.	**Distance matrix of local breeds with geographic distance as covariate**	
**Partial Mantel test**	**r**_**M**_	**p-value**
*Human population distance*	0.318	<0.001
*Ecological distance*	0.225	<0.001
*Historical distance*	0.198	0.002

Significance was assessed using 1000 randomizations. (b) Results of the Partial Mantel test (r_M_ and p-values) of the distance matrix of local breed and the rest of distance matrices (human population, ecological, historical) using the geographic distance as covariate matrix.

**Figure 1:**
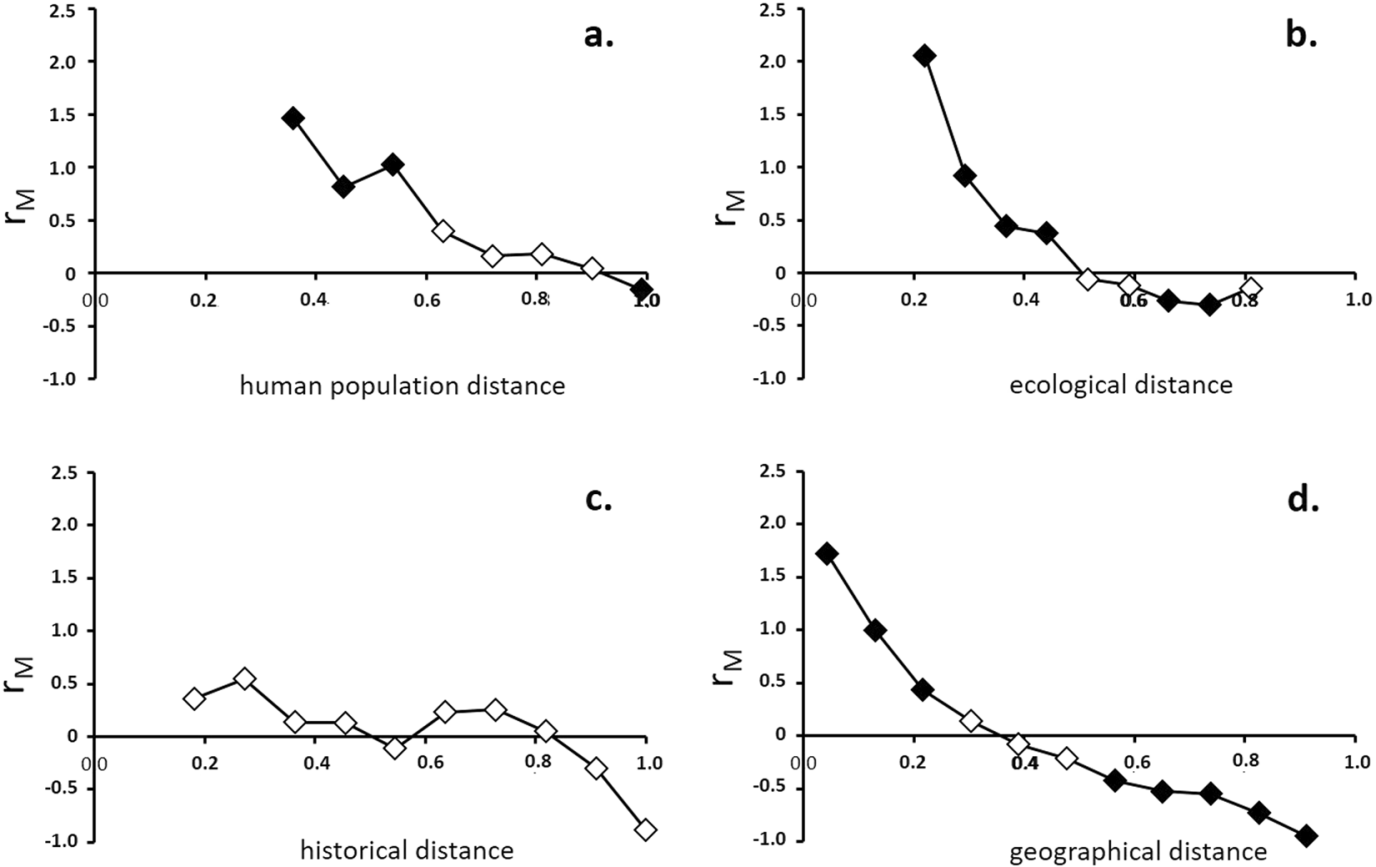
Partial Mantel correlograms showing the structuring of the provincial composition of local breeds within 11 classes of different distances: (**a**) human population distance; (**b**) ecological distance (based on vertebrate animals); and (**c**) historical distance. The geographic distance matrix was included as the covariable matrix. Correlogram (**d**) of geographical distances is a simple Mantel correlogram with the spatial structure of the provincial composition of local breeds within the different distance classes. The midpoint of each distance class is plotted. Black diamonds show significant correlation after Bonferroni correction.

Partial Mantel correlograms using geographical distance as the covariate matrix are shown in Fig. 1. Mantel tests were carried out on each of the 11 distance classes following the Sturge’s rule. In some cases, the first distance classes were not testable (null result for P and r_M_ values) because they contained no variation: the first three classes for the correlogram of local breed variation by human population distances, the first two for the correlogram of ecological distances and the first of the one of historical distances. According to the partial Mantel correlograms, the provincial composition of local breeds shows isolation by human population distance (Fig. 1a) and isolation by ecological distance (Fig. 1b). That is, the more similar two provinces are in human and environmental terms, the more similar their local breeds are. Local breeds did not show a clear pattern of isolation by historical distances, with no significant p-values (Fig. 1c). On the contrary, the composition of local breeds responds to a model of isolation by geographical distance (simple Mantel correlogram) (Fig. 1d). In the correlogram of the human population, four of the eight distances classes showed significant Bonferroni corrected p-values. Six of nine were significant for the correlogram of ecological distances, as were eight of 11 in the case of the correlogram of geographical distances.

**Figure 2:**
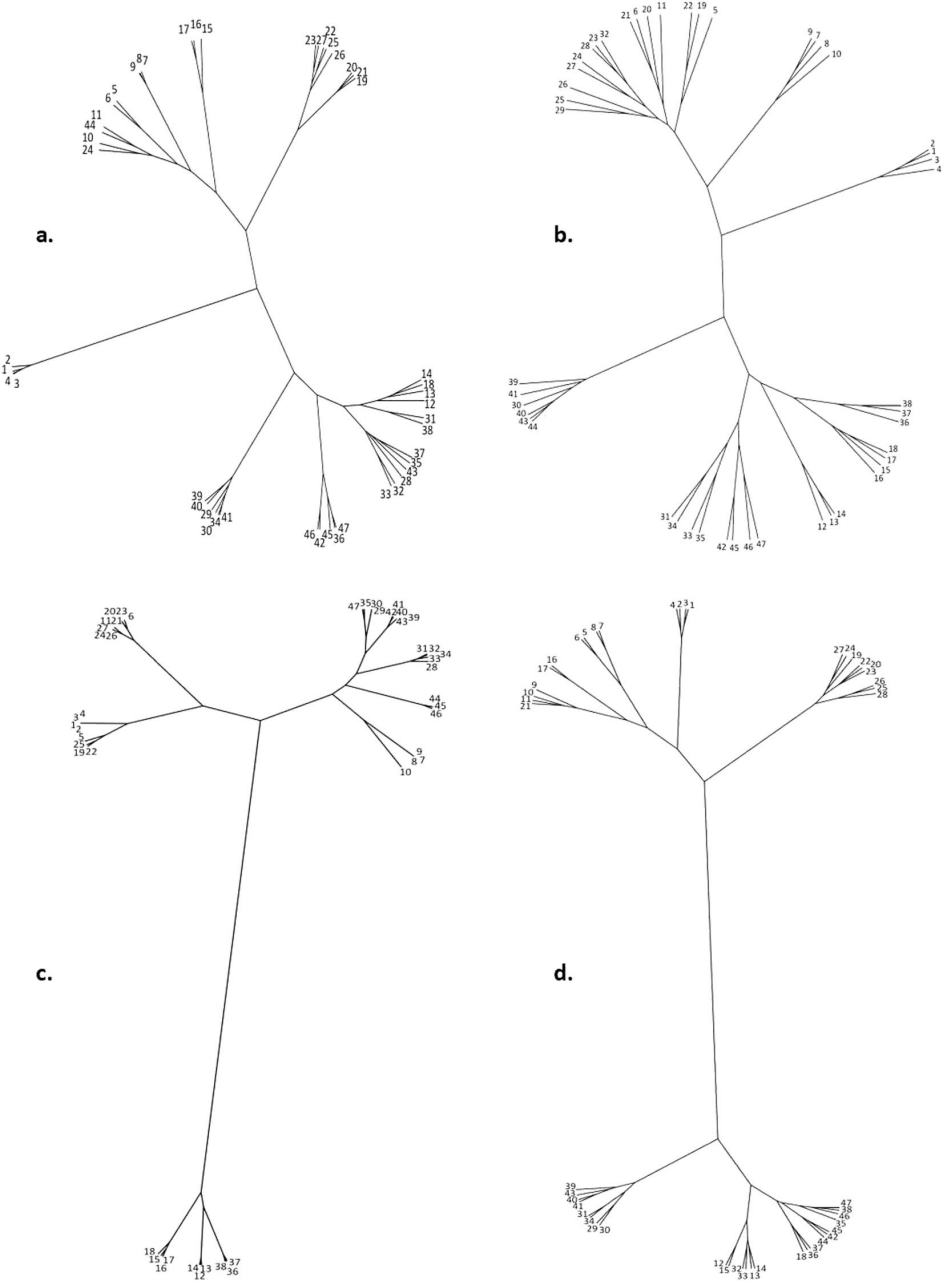
Unrooted, neighbor-joining tree showing the affiliations of mainland Spain provinces in relation to: (**a**) local breeds; (**b**) human population; (**c**) ecological characteristics; and (**d**) historical ties. Correspondence between cluster numbers and mainland Spain Provinces: *La Coruña* (1); *Lugo* (2); *Pontevedra* (3); *Orense* (4); *Asturias* (5); *Cantabria* (6); *Vizcaya* (7); *Guipúzcoa* (8); *Álava* (9); *Navarra* (10); *La Rioja* (11); *Huesca* (12); *Zaragoza* (13); *Teruel* (14); *Lérida* (15); *Gerona* (16); *Barcelona* (17); *Tarragona* (18); *León* (19); *Palencia* (20); *Burgos* (21); *Zamora* (22); *Valladolid* (23); *Soria* (24); *Salamanca* (25); *Ávila* (26); *Segovia* (27); *Madrid* (28); *Cáceres* (29); *Badajoz* (30); *Toledo* (31); *Guadalajara* (32); *Cuenca* (33); *Ciudad Real* (34); *Albacete* (35); *Castellón* (36); *Valencia* (37); *Alicante* (38); *Huelva* (39); *Sevilla* (40); *Córdoba* (41); *Jaén* (42); *Cádiz* (43); *Málaga* (44); *Granada* (45); *Almería* (46); *Murcia* (47). (Supplemental Figure S1 includes a map with the provinces of mainland Spain. See Supplementary Data).

Figure 2 shows the unrooted cluster diagrams depicting the relationships among the provinces of mainland Spain regarding local breeds, human population, ecological characteristics and historical ties.

The multiple matrix regression with the distance matrix of local breeds as response matrix (R^2^ = 0.36, p < 0.001) showed that the distance matrix of the human population had a higher regression coefficient. The effects of ecological, historical, and geographical distance are also significant, although their coefficients are lower (Table 2).

**Table 2:** The result of the multiple matrix regression with the distance matrix of local breeds as response and human population, ecological, historical and geographical distance matrices as explanatory variables.

Matrices	r_M_	p-value
*Intercept*	0.232	1.00
*Human population distance*	0.292	0.01
*Ecological distance*	0.195	0.01
*Historical distance*	0.108	0.02
*Geographic distance*	0.139	0.02

## Discussion

We found that the more similar two provinces of mainland Spain are in terms of human population, ecological characteristics, historical ties, and spatially close, the more similar will be the composition of local breeds in their territories. Thus, we provide empirical evidence to support the view of local breeds as products of complex cultural and environmental processes responding to both natural and artificial selection.

The highest standardized coefficient for the human population distances in the multiple matrix regression underpins the importance of the human background in livestock niches. The spatial structure of local breeds is connected with human migration patterns and interchanges among neighboring human populations. Related populations tend to share similar breeds. Within a human group, local breeds and people have been interrelating and co-evolving for many centuries (at least in the Old World), and diverging from other human groups. For this reason, areas with high human cultural diversity also host high levels of livestock biodiversity, as has been shown for the Old World^42^. There are many examples in the literature of the close links between local breeds and human groups, although these tend not to be statistically quantified. In Sicily, the human population is divided geographically and genetically into two groups, each of which manages its own native breed of cattle^1*based on*^ ^43,44^. The spatial distributions of the two yak breeds in Sichuan (China) overlap with the Kham and Anido ethnic groups respectively^45^. Herdwick sheep and people from Lake District (north-west England) share an ancient Scandinavian past^11^. The only studies where the relationship between humans and domesticated species has been explicitly quantified was carried out by Tanabe^46^, who found that genetic similarities between Japanese dog breeds are paralleled by genetic affinities between the corresponding human groups.

It was also found that historical distances are involved in local breed distribution. Historical influence can be manifested in several ways. Political boundaries can condition population movements, but surname distances include this fact (for the last seven centuries). At the same time, this historical sequence of borders may have imposed limitations on breed flows and interchanges between territories. Moreover, each political unit develops their own laws and their own economic guidelines. In addition, although sociological and anthropological studies have shown that political units and culture are not necessarily isomorphic, borders represent relevant instruments within the dialectic of identity and otherness, promoting belonging and connectedness among the community members^47^. All of these reasons could explain the role of political borders in breed spatial distribution. Local breeds might even strengthen community cohesion. Accordingly, several breeds have achieved the recognition of symbols of a certain country or region.

Other cultural factors not encompassed in the analyses, such as linguistic differences, might also contribute to understanding the spatial variations in livestock niches. Indeed, the Spanish regions that conserve their own languages, such as Galicia, Basque Country or Catalonia, form distinct groups within the local breed cluster shown in Fig. 2a.

Nevertheless, whatever the objectives pursued in artificial selection, they will always be limited by the fact that, ultimately, breeds have to be able to successfully cope with local specific environmental conditions. Natural selection pressures, therefore, are also relevant. In this study, ecological distances showed the second highest regression coefficient within the multiple matrix regression. That means that the environmental factors that affect the spatial distribution of biodiversity are also involved in the diversity distribution of local breeds. Hence, differences in the environmental conditions require ecological adaptations which include changes in the anatomy, morphology, physiology, feeding behavior, metabolism, or performance^48^. These adaptations to a wide variety of environmental extremities have allowed livestock expansion worldwide^49^. However, although indigenous breeds are assumed to be locally adapted, further research is needed to explain how certain breeds are adapted to a given environment and in which other environments they can survive, especially in climate change context^50^. For example, some responses to climatic challenges and disease resistance have been described at the genome level in African cattle^51,52^.

Geographical distances are also relevant, suggesting that spatial proximity has guided the relation between local breeds. This model of isolation by distance has already been described in livestock at the genetic level. Thus, the genetic structure of European breeds is consistent with their geographical origin^17^. A correlation between the genetic distances among breed groups and their geographical locations was found using a phylogenetic tree of 216 cattle breeds from polymorphism records^53^. Kantanen *et al*.^10^ showed that the Icelandic cattle breed was more genetically related to the nearest Scandinavian breeds. The own methodologies applied to the search for signatures of selection take into account the isolation by distance^21^. Nevertheless, this relationship is not always true. The genetic distances among 15 cattle breeds of Spain and Portugal showed no correlation with the geographical distances between their traditional centers of distribution (Hall^1^, using data from Cañon *et al*.^54^ and Porter^55^).

All factors together explaining the spatial structure of local breeds support the idea of livestock niche as an environmentally mediated socio-cultural construction, which corresponds to the three models of spatial cultural variation proposed by Guglielmino *et al*.^56^. In accordance with these authors, cultural traits evolve by demic diffusion, cultural diffusion, and ecological adaptation. This third model of cultural variation, the adaptation to environmental constraints, is an essential mechanism in shaping livestock niches. In fact, both humans and animals not only adapt but also contribute actively to modify their ecological conditions, thereby generating a transformed landscape that benefits both, and which contributes to its long term maintenance. This transformed landscape represents the territorial expression of the livestock niche. In this process of niche construction, humans make good use of all of the traditional ecological knowledge inherit from their ancestors. This ecological knowledge is transmitted through stories, rites, myths, and symbols, frequently converted into elements of the environment^57^. Local breeds, transformed landscapes, and ecological knowledge are part of the ecological inheritance socially transmitted through generations^39^ and embodied in the livestock niche. However, ecological inheritance is not culturally neutral because humans operate based on their beliefs and systems of values. Cultural inheritance also engages in the niche construction^58^. What might happen is that the environment imposes a number of restrictions within which culture can operate. On the other hand, livestock niches are in continuous evolution and update as a result of adaptive processes, either by internal innovation or by external influence. Changes from outside the group arrive through the cultural transmission of innovations following the other two models of cultural variation. Demic diffusion is related to human migrations and radiation in which migrants take with them their culture and beliefs to these new areas, probably also including local breeds. The footprints of these human movements are tracked within the surname spatial structuring, at least for the last eight centuries. By cultural diffusion, one human group acquires cultural traits from the neighboring groups. Although specific indicators of cultural diffusion were unavailable, it is expected that cultural flows will be greater among those territories with lively population interchanges^59,60^, which would be reflected in the surname distribution. In addition, the historical administrative divisions have restricted cultural flows across their borders, while at the same time encourage the internal ones. We acknowledge that the time range covered by the analysis does not incorporate relevant processes of demic and cultural diffusion related to livestock prior to the appearance of surnames in the 13^th^ century. Phylogenetic data shows that livestock accompanied humans in their migrations throughout the last millennia^20^, which has also been described for Spain^61^.

In their study for Sub-Saharan societies, Guglielmino *et al*.^56^ also found that livestock traits were related to cultural inheritance accumulated within the own human group and the adoption of practices from neighboring groups, but also to ecological factors. Under this approach, processes as selective breeding mutation, adaptation, and isolation or genetic drift, all implicated in the creation of the wide diversity of local breeds^18^, can be seen as embedded in an evolutionary framework based on a triple inheritance: genetic, cultural, and ecological^32,62^.

It is possible to highlight some lessons from our findings. In terms of Cavalli-Sforza & Feldman^63^, the system of socio-cultural transmission of traditional ecological knowledge favors livestock niche preservation. In the demic model, the highly conservative vertical and group pressure mechanisms of cultural evolution are predominant. Moreover, the environmental conditions and the engineered ecosystems resulting from ecological adaptation also impose constraints to innovation. As a result, both cultural and ecological inheritances make it increasingly difficult for new generations of the human group to abandon the cultural practices received from their ancestors^38^. This conservative approach helps to ensure long-term local breed preservation but at the same time could slow down an adaptive response to new circumstances. This procedure contrasts sharply with the speed of changes linked to the processes currently driving the evolution of livestock niches, climate change, and globalization. These two processes of rapid change are probably modifying the environmental and cultural factors related to livestock niche construction. For example, climate change is imposing a new process of ecological adaptation. Breeders can address the impacts on livestock niches by the artificial selection of animal traits more adapted to the new conditions or, more likely at first, by the inclusion of modifications in husbandry practices^14,50,64^. The pressures of globalization by processes of long-distance cultural diffusion impose the replacement of local breeds by commercial breeds according to criteria of economic profitability. Indeed, human groups closely linked to a certain local breed could switch to another if circumstances require so^40^.

The extinction of a local breed reduces the genetic wealth available to face the changing environment and emerging diseases^65^ and also might lead to the disappearance of a unique livestock niche resulting from the cultural and ecological inheritances accumulated over the years in the territory by the human-animal relationship. Part of the identity of people would disappear with the loss of a local breed, although the importance of the associated cultural values shows relevant differences among breeds^66–68^. The necessary investments to ensure long-term conservation of local breeds will be affordable only in certain cases. However, together with economic efficiency and international efforts to conserve them, the domestic preservation of a certain local breed will depend on how its related human group resolves the local/global tensions. The recent rise of local identities against the homogenization imposed by globalization may favour the conservation of local breeds.

## Material and Methods

### Area of study

The study was conducted in mainland Spain. Spain has an extension of 492,175 km^2^_,_ and its average height is 660 m above sea level. Spain is positioned between the Atlantic Ocean and the Mediterranean Sea, making it a true biogeographic and cultural crossroad. The central area of the peninsular territory is occupied by a large plateau surrounded by several mountainous ranges.

Both the Atlantic Ocean and the Mediterranean Sea regulate the climate of the Iberian Peninsula; the Atlantic provides moisture and moderate temperatures (≈14 °C), and the Mediterranean coast is characterized by dry summers and higher temperatures (15–18 °C). The inland regions show marked continental characteristics with annual average temperatures ranging from 10 °C in the North to more than 16 °C in the South. Within this mainland territory, three biogeographic regions of the seven existing in the European Union are found: Atlantic, Mediterranean, and Alpine.

The human population in mainland Spain is about 43 million, distributed across 47 provinces (15 autonomous regions). Coastal areas are highly populated compared to the center of the country (with the exception of the capital, Madrid).

Since the beginning of the surname system (progressively consolidated between the 13^th^ to the 15^th^ century), the political configuration of mainland Spain has varied over time. The period between the 13^th^ century and 1492 is part of the Reconquista, during which Christian Iberian kingdoms placed in northern Spain conquered the territories ruled by the Arabs in the South. Despite the dynastic union, Castile and Aragon retained separate legal systems and their own administrative divisions until the Nueva Planta decrees at the beginning of 18^th^ century. In this century, Spain was divided into intendants. Subsequently, the territorial division defined in 1833 divided the Spanish territory into provinces, which have remained virtually unchanged until today, although there had been multiple attempts of regionalization (usually based on “historical regions”) until the actual division in Autonomous communities created in accordance with the Spanish Constitution of 1978.

Finally, there are four different languages; Spanish, which is the official language in the whole territory; Catalan, spoken in the coastal area of Eastern Spain; Galician, in the Northwest corner; and Euskera, in the North.

### Livestock breed in Spain

Livestock farming contributes around 40% of the agricultural output in Spain. This livestock production has become progressively more dependent on commercial breeds to the detriment of local ones, following the same trend as in other parts of the world. Nevertheless, traditional production systems based on the use of local breeds adapted to the environment coexist with modern forms of intensive production using the most advanced technologies. The Spanish Official Catalogue of Livestock Breeds (SCLB) currently lists 181 breeds, including 153 local breeds, of which 126 are classified as endangered or facing extinction. The exact origin of many extant breeds is not well known.

As regards spatial distribution, cattle predominate in humid regions with abundant grass. Sheep are more widely distributed throughout the country. Goats traditionally have occupied mountainous areas. Pigs are related to the dehesas, the agrosilvopastoral system of the western and southwestern Spain. Horses and donkeys are also more common in the West.

### Data sources

Livestock breed data was obtained from the Spanish National Information System of livestock breeds (ARCA System), which contains updated information about the breeds included in the SCLB. The ARCA system is managed by the Ministry of Environment in cooperation with the regional governments and breeders’ associations, with the aim of maintaining a complete inventory of Spanish animal genetic resources, population trends, and associated risks. The ARCA database contains the number of livestock heads for each breed in each Spanish province. Only local breeds with a presence in mainland Spain were considered in the study. Although the proportion of commercial breeds within the total stock of livestock has increased considerably in last decades, many of them have been introduced recently, are reared in intensive farms with little interaction with the local environment, and, therefore, play a limited role in the historical construction of livestock niches. Breeds of poultry were also removed from the final dataset because their provincial censuses were not available. A total of 105 breeds were included: 34 cattle, 34 sheep, 14 goat, 11 horse, 3 donkey and 9 pig breeds.

The surname database was obtained from the Continuous Register of Population at 2008, managed by the Spanish National Institute of Statistics. The dataset is organized by municipalities and includes, for each municipality, the number of times that each surname with at least 5 records appears in that place. Both paternal and maternal surnames were considered, which increases the sample size and the strength of the analyses^69^. The initial dataset included a total of 56,976,706 records of surnames. The number of different surnames was 87,148. Obvious misspelling and digitalization errors were corrected. Surnames were grouped by their spatial distribution among Spanish provinces using self-organizing maps^70,71^. Since this procedure based on unsupervised neural networks groups surnames with a similar frequency in each province, it enables us to use surnames as geographical markers to characterize the historical relationships among the Spanish human population. This procedure also enables us to identify polyphyletic surnames, which were removed from the analyses. Only those surnames with more than 20 records and with a clear geographical origin were included. A more detailed description of the methodology can be found in Manni *et al*.^72^ and Rodriguez-Diaz *et al*.^73^.

The sequence of political divisions in which Spanish territory has been divided since the 13^th^ century was obtained from the Historical Atlas of Spain (2004). That century was chosen as the starting point for the historical variable so as to have the same time range as the surnames, which emerged at that time.

The spatial distributions of vertebrate species were obtained from the Spanish Inventory of Terrestrial Biodiversity promoted by the Ministry of Agriculture, Food, and Environment. The inventory compiles information about species spatial distributions contained in the various Atlases and Red Books. This information is regularly updated with new data provided by the monitoring programs of each taxonomic group. Spatial distributions are shown in a 10 × 10 km UTM grid covering the entire Spanish territory. We only considered terrestrial vertebrates since their distributions are better known and have lower biases caused by different sampling intensities between zones. Thus, we worked with 27 species of amphibians, 256 of birds, 87 of mammals, 38 of freshwater fishes, and 42 of reptiles; a total of 450 species.

### Distances among Spanish provinces

Since the Spanish ARCA System for livestock breeds compiles local breed censuses at the provincial level, the unit of the study was the province (n = 47). Square matrices of pairwise distances between the 47 provinces for local breeds, surnames, history, vertebrate species of wildlife, and geography were obtained (Supplementary Material: Tables S1–S5, respectively). Distances were Euclidean distances in all cases. Pairwise local breed distances between all provinces were assessed by comparing the percentages of heads of each breed in relation to the total number of heads within its group of livestock (cattle, sheep, goats, horses, donkeys, and pigs) in that province. Provinces with similar local breeds and in similar percentages within each group will be close, and vice-versa. Pairwise surname distances (*human population distances*) were obtained from the comparison of the surnames present in each province. Surnames were used to quantify the relationship between human populations. Considering the surnames as geographic markers, it is expected that the more similar the surnames between two provinces, the greater the relationship and the population exchange between both. Pairwise *historical distances* were calculated considering the belonging of each province to the different political divisions in Peninsular Spain since the 13^th^ century (origin of the Spanish surnames) and at intervals of 100 years. Pairwise distances between provinces in relation to vertebrate species distributions (*ecological distances*) were obtained by comparing the percentage of positive 10 × 10 UTM grids in each province for the 450 vertebrate species according to the distributions contained in the Spanish Inventory of Terrestrial Biodiversity. Pairwise *geographical distances* were calculated based on the linear distance between province centroids.

### Statistical analysis

The Mantel test^74^ was performed to identify the correlation between all distance matrices. To rule out that the results correspond to a mere isolation model by distance, partial Mantel tests were performed. Partial Mantel analysis calculates the correlation between two distance matrices controlling for the effect of a third matrix. Thus, the one-to-one correlation between the distance matrix of local breeds and the distance matrices of human population, history, and ecological characteristics were calculated considering the effect of geographical distances. For this purpose, a geographic distance matrix was used as the covariable matrix. Ten-thousand permutations were used in significance testing in both the Mantel and partial Mantel tests. Partial Mantel correlograms controlling for the effect of geographical distances were constructed following the specifications exposed by Legendre and Legendre^75^. Correlograms quantify how inter-site similarity varies with inter-site distance and are frequently used to describe spatial patterns^76^. Partial Mantel correlogram performs a Mantel test on different distance classes and generates a correlogram with the Mantel test statistic on the y-axis obtained for each distance class. Distance classes are represented on the x-axis. Correlogram shape can be used to define the underlying structure that exists between the two input distance matrices. Sturge’s rule was followed to determine an appropriate number of distance classes. Bonferroni correction for multiple comparisons was applied^75^.

In addition, multiple regression on distance matrices (MRM) was used to estimate the independent effects of the human population, historical and ecological matrices (explanatory matrices) on the breed matrix (response matrix). MRM provides inferences about the relationships between distances by a multiple regression of a response distance matrix on two or more distance matrices, where each matrix contains distances between all pair-wise combinations of the elements of a set (here, provinces of mainland Spain). Tests of statistical significance for regression coefficients and R-squared are performed by permutation^77^. Here, the analysis was implemented with 10,000 permutations. The “ecodist” package was used to carry out the analyses^78^. Moreover, cluster analysis was performed to explore the relations among provinces in relation to local breeds, human population, ecological characteristics and historical ties. Provinces were grouped by hierarchical agglomerative clustering using Ward’s method performed on the distance matrices. Packages used were “stats”^79^ and “ape”^80^.

## Electronic supplementary material

Figure S1

Tables S1-S6
