# Supplementary material for: Human and ecological determinants of the spatial structure of local breed diversity
Source: Sci Rep. 2018 Apr 24;8:6452. doi: 10.1038/s41598-018-24641-3 (PMC5915451; doi:10.1038/s41598-018-24641-3)

**Human and ecological determinants of the spatial structure of local breed diversity**

Victor J Colino-Rabanal^1^*, Roberto Rodríguez-Díaz^2^, María José Blanco-Villegas^2^, Salvador J Peris^1^, Miguel Lizana^1^

^1^ Area of Zoology. Dept. of Animal Biology, Parasitology, Ecology, Edaphology and Agronomic Chemistry. University of Salamanca. Campus Miguel de Unamuno. 37071 Salamanca, Spain.

^2^ Area of Physical Anthropology. Dept. of Animal Biology, Parasitology, Ecology, Edaphology and Agronomic Chemistry. University of Salamanca. Campus Miguel de Unamuno. 37071 Salamanca, Spain.

*Corresponding author

Victor J Colino-Rabanal

E-mail: [vcolino@usal.es](mailto:vcolino@usal.es)

Phone: 34-676643770

e-mail Roberto Rodríguez-Díaz: [roberrd@usal.es](mailto:roberrd@usal.es)

e-mail María J Blanco Villegas: mache@usal.es

e-mail Salvador J Peris: [peris@usal.es](mailto:peris@usal.es)

e-mail Miguel Lizana: lizana@usal.es

**Figure S1.** Map with the 47 mainland Spain Provinces. The correspondence between numbers and provinces is: La Coruña (1); Lugo (2); Pontevedra (3); Orense (4); Asturias (5); Cantabria (6); Vizcaya (7); Guipúzcoa (8); Álava (9); Navarra (10); La Rioja (11); Huesca (12); Zaragoza (13); Teruel (14); Lérida (15); Gerona (16); Barcelona (17); Tarragona (18); León (19); Palencia (20); Burgos (21); Zamora (22); Valladolid (23); Soria (24); Salamanca (25); Ávila (26); Segovia (27); Madrid (28); Cáceres (29); Badajoz (30); Toledo (31); Guadalajara (32); Cuenca (33); Ciudad Real (34); Albacete (35); Castellón (36); Valencia (37); Alicante (38); Huelva (39); Sevilla (40); Córdoba (41); Jaén (42); Cádiz (43); Málaga (44); Granada (45); Almería (46); Murcia (47). The map was created from the shapefile of Spanish Provinces included in the National Topographic Base at a 1:500 000 scale provided by «© Instituto Geográfico Nacional». QGIS 2.18.2 ([www.qgis.org](http://www.qgis.org)) was used to generate the map.


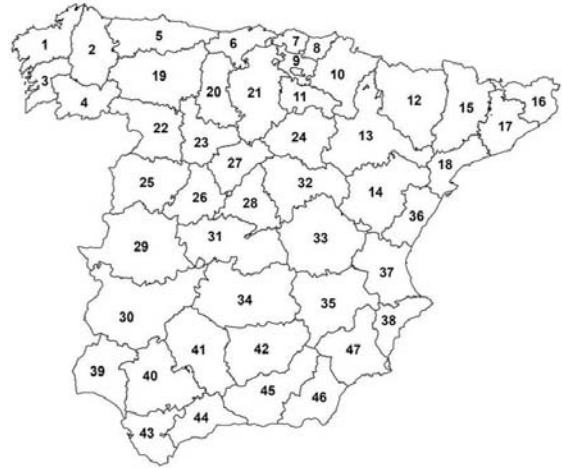

Supplement: Supplementary file 1 — Figure S1 [file 41598_2018_24641_MOESM1_ESM.docx]
